# Comparison of serum lipid management between elderly and non-elderly patients with and without coronary heart disease (CHD)

**DOI:** 10.1016/j.pmedr.2016.06.006

**Published:** 2016-06-08

**Authors:** Rumiko Shimizu, Haruki Torii, Daisuke Yasuda, Yoshinori Hiraoka, Yutaka Furukawa, Akihiro Yoshimoto, Toshio Iwakura, Naoki Matsuoka, Keisuke Tomii, Nobuo Kohara, Tohru Hashida, Noriaki Kume

**Affiliations:** a Division of Clinical Pharmacy, Faculty of Pharmaceutical Sciences, Kobe Gakuin University, 1-1-3 Minatojima, Chuo-ku, Kobe 650-8586, Japan; b Department of Cardiology, Kobe City Medical Center General Hospital, 2-2-1 Minatojimaminami-machi, Chuo-ku, Kobe 650-0047, Japan; c Department of Nephrology, Kobe City Medical Center General Hospital, 2-2-1 Minatojimaminami-machi, Chuo-ku, Kobe 650-0047, Japan; d Department of Diabetes and Endocrinology, Kobe City Medical Center General Hospital, 2-2-1 Minatojimaminami-machi, Chuo-ku, Kobe 650-0047, Japan; e Department of Respiratory Medicine, Kobe City Medical Center General Hospital, 2-2-1 Minatojimaminami-machi, Chuo-ku, Kobe 650-0047, Japan; f Department of Neurology, Kobe City Medical Center General Hospital, 2-2-1 Minatojimaminami-machi, Chuo-ku, Kobe 650-0047, Japan; g Department of Pharmacy, Kobe City Medical Center General Hospital, 2-2-1 Minatojimaminami-machi, Chuo-ku, Kobe 650-0047, Japan

**Keywords:** Coronary heart disease (CHD), Elderly, LDL-cholesterol, Lipid-lowering medication, Non-HDL-cholesterol

## Abstract

Serum lipid management in patients aged ≥ 75 has not been precisely explored. We, therefore, compared the serum lipid management between the two age groups with and without coronary heart disease (CHD).

We, therefore, retrospectively reviewed medical charts of patients who were hospitalized in the departments of internal medicine during a period of 14 months. Serum lipid goal attainment was explored by applying the lipid goals for patients aged < 75 to those aged ≥ 75.

In 1988 enrolled patients, 717 subjects (36.1%) were aged ≥ 75. Among them, 41.3% and 32.4% of the patients had CHD, 44.2% and 41.0% were primary prevention at high-risk, and 14.5% and 14.6% were primary prevention at moderate-risk in patients aged ≥ 75 and aged < 75, respectively. Serum LDL-C goal achievement rates in CHD were 66.9% and 65.0% in patients aged ≥ 75 and < 75, respectively (*p* = 0.334). In the primary prevention at high-risk, these rates were 73.5% and 63.3%, in patients aged ≥ 75 and < 75, respectively (*p* = 0.001). They were 77.9% and 58.1% in primary prevention at moderate-risk aged ≥ 75 and < 75, respectively (*p* < 0.001). In CHD, lipid-lowering medication subscription rates were significantly lower in patients aged ≥ 75 (60.1%) than those aged < 75 (73.8%, *p* < 0.001).

In conclusion, in CHD, serum lipid goal attainment was comparable between the two age groups although the lipid-lowering drugs were less frequently prescribed in patients aged ≥ 75. Without CHD, it was significantly better in patients aged ≥ 75 than those aged < 75 although the lipid-lowering drug subscription rates were comparable between the two age groups.

## Highlights

• Serum lipid goal attainment was comparable between patients aged ≥ 75 and < 75 with coronary heart disease (CHD).
• Serum lipid goal attainment was better in patients aged ≥ 75 than those aged < 75 without CHD.
• Lipid-lowering medication was less frequently prescribed in patients aged ≥ 75 than those aged < 75 with CHD.
• Lipid-lowering medication was almost equally prescribed between patients aged ≥ 75 and < 75 without CHD.
• Statin plus EPA was less frequently prescribed in patients aged ≥ 75 than those aged < 75 with or without CHD.

## 1 Introduction

The incidence and prevalence of atherosclerotic cardiovascular disease (ACVD) increase with age (de Ruijter et al., 2009, Berthold and Gouni-Berthold, 2011, Phan and Bittner, 2014, McDermott, 2007, Petersen et al., 2005, Rosamond et al., 2007), and the majority of ACVD events occur after age 70 years (Stone et al., 2014). In 2009, the annual mortalities from acute myocardial infarction per 100,000 Japanese population were 12.4 and 18.4 in people aged 50 to 54 years and 55 to 59 years, respectively. These rates were 127.8 and 215.0 in the older people aged ≥ 65 years and ≥ 75 years, respectively. Thus, more than 10-fold higher ACVD mortality was observed in the elderly (≥ 75) age group when compared to the middle (50 to 59) age group (Japan Atherosclerosis Society, 2014). Because demographic aging is proceeding at an unprecedented speed in Japan, the incidence for ACVD is also predicted to be increasing. Dyslipidemia, especially the high LDL cholesterol (LDL-C) level, is one of the most important risk factors for ACVD; therefore, management of LDL-C is extremely important for preventing ACVD in the older population. However, the importance of dyslipidemia as an ACVD risk factor in older adults appeared controversial (Ettinger et al., 1992). Several studies have suggested that the association between cholesterol levels and ACVD weakens with age and that there may be little potential benefit from screening and treating older patients with dyslipidemia (Gordon and Rifkind, 1989, Mariotti et al., 1986, Garber et al., 1991). Conversely, some investigators have shown that cholesterol concentrations retain a significant risk factor for ACVD in the elderly (Benfante and Reed, 1990, Barrett-Connor et al., 1984, Rubin et al., 1990), and lowering serum cholesterol in the elderly may have a greater impact on ACVD than in the middle age people because the absolute attributable risk of ACVD from dyslipidemia is greater in the older age group than in the middle age group, although the relative risk of ACVD derived from dyslipidemia is smaller in the older age group than in the middle age group.

The Japan Atherosclerosis Society guidelines for prevention of atherosclerotic cardiovascular diseases 2012 (JAS2012-GL) suggest the following: Subjects with dyslipidemia whose ages are between 65 and 74 should be treated in the same way as those aged below 65 to achieve their serum lipid goals. In cases of subjects with dyslipidemia whose ages are no less than 75 (≥ 75), patients with primary prevention for coronary heart disease (CHD) can be treated individually by the specific decision of the attending physician, although dyslipidemic patients with secondary prevention for CHD should be treated equally to those whose ages are below 65 to achieve their serum lipid goals (Japan Atherosclerosis Society, 2014). We, therefore, anticipated that the lipid goal attainment in CHD (secondary prevention) may be similar between patients aged ≥ 75 and < 75 and that it may be better in patients aged < 75 than in those aged ≥ 75 whose serum lipid control may not be mandatory in some cases.

Thus, to examine whether patients with dyslipidemia aged ≥ 75 (the elderly group) are treated differently from those aged < 75 (the non-elderly group), serum lipid goal achievement rates were compared by applying the lipid goal for the patients aged < 75 to those aged ≥ 75 by use of the JAS-GL2012. In addition, those rates were further compared between the two age groups in different risk category subgroups, such as high-risk and moderate-risk patients with primary prevention for CHD and those with secondary prevention for CHD. Furthermore, contents of lipid-lowering medication were compared between the elderly and non-elderly groups.

## 2 Methods

### 2.1 Study population

Medical charts of all the patients who were hospitalized in the Departments of Nephrology, Diabetes, Neurology, Respiratory Medicine and Cardiology, at Kobe City Medical Center General Hospital, Kobe, Japan, from April 1st, 2012 to May 31st, 2013 were retrospectively reviewed. This hospital has 700 beds, which comprises approximately 4.6% of the total number of the hospital beds (15,367) in Kobe City, whose population is 1,535,037. Subjects who underwent regular dialysis because of chronic renal failure or without serum lipid data were excluded. Chronic kidney disease (CKD) at the stage III or higher, according to the guideline from the Japanese Society of Nephrology, was regarded as a high risk for ACVD. Diabetes mellitus (DM) was diagnosed according to the guideline from the Japan Diabetes Society. CHD was defined as myocardial infarction, angina pectoris, or history of percutaneous coronary intervention (PCI) or coronary artery bypass graft (CABG) surgery which was described in the medical chart. We have assessed their medical conditions by reading the medical chart one by one, and confirmed them by, at least, two different investigators. LDL-C levels were calculated by Friedewald's formula. When serum TG levels were above 200 mg/dL, measured values of LDL-C by a direct LDL-C measurement kit from Sekisui Medical Co. Ltd. were utilized. Direct measurement of LDL-C was performed in the clinical laboratory at the hospital as a part of clinical practice. When lipid levels were evaluated more than once, their steady state levels after admission were utilized. In this study, CKD group (CKD-G) did not include CKD with CHD or DM, and DM group (DM-G) did not contain DM with CHD, because of the risk stratification under the JAS-GL. This study protocol has been approved by the ethical committees in Kobe City Medical Center General Hospital and Kobe Gakuin University.

### 2.2 Statistical analysis

Continuous variables are presented as mean ± standard error of mean (SEM), and categorical variables are shown as percentages and numbers. Continuous variables were compared using the Student's *t*-test and Welch's *t*-test, if the Levene test showed the equal and unequal variance, respectively. The significance in the differences for categorical variables was determined by the χ^2^ test. Moreover, supplementary residual analysis was performed for comparisons of more than two categories. All statistical analyses were carried out using IBM SPSS Statistics 23 (SPSS Inc.). *P* values below 0.05 (*p* < 0.05) were considered as statistically significant.

## 3 Results

### 3.1 Patient enrollment

Medical charts of all the 3785 patients who were hospitalized in the Departments of Nephrology, Diabetes, Neurology, Respiratory Medicine and Cardiology at Kobe City Medical Center General Hospital, from April 1st, 2012 to May, 31st, 2013 were retrospectively reviewed. Sixteen hundred and sixty (1660) subjects without lipid data, as well as 137 patients who underwent regular dialysis because of chronic renal failure, were excluded. As a result, a total of 1988 patients were enrolled. The numbers of patients who were enrolled from Departments of Nephrology, Diabetes, Neurology, Respiratory Medicine and Cardiology were 180, 176, 41, 277 and 1314, respectively.

### 3.2 Patient characteristics

Characteristics of enrolled patients are summarized in Table 1. There was a significant difference in the proportion of patients aged ≥ 75 (overall: *p* < 0.001), due to the higher prevalence of patients aged ≥ 75 in Department of Respiratory Medicine (*p* < 0.01) and the lower prevalence of those in Departments of Nephrology (*p* < 0.05) and Diabetes (*p* < 0.01). BMI (*p* < 0.001), all lipid levels (*p* < 0.001) and eGFR (*p* < 0.001) were significantly lower in patients aged ≥ 75 than those aged < 75. In addition, the prevalence of female (*p* < 0.001), HT (*p* = 0.001), CKD (*p* < 0.001) and CHD (*p* < 0.001) was significantly higher in patients aged ≥ 75 than those aged < 75.

### 3.3 Comparison of LDL-C and non-HDL-C levels and their target level achievement rates between male and female

To explore whether the gender imbalance between patients aged ≥ 75 and < 75 can be the cause for the differences in lipid levels between the two age groups, lipid profiles were compared between male and female. As shown in Table 2, LDL-C (*p* < 0.001), HDL-C (*p* < 0.001) non-HDL-C (*p* < 0.001) levels were significantly higher in female than in male. However, TG (*p* < 0.001) level was significantly lower in female than in male. LDL-C target level achievement rates were 68.4% and 66.8%, in male and in female, respectively (*p* = 0.255). These rates for non-HDL-C were 70.8% and 70.3%, respectively (*p* = 0.427). Thus, lipid target level achievement rates were comparable between male and female, although there were significant differences in lipid levels.

### 3.4 Comparison of risk stratification profiles between the elderly and the non-elderly age groups

**Fig. 1:**
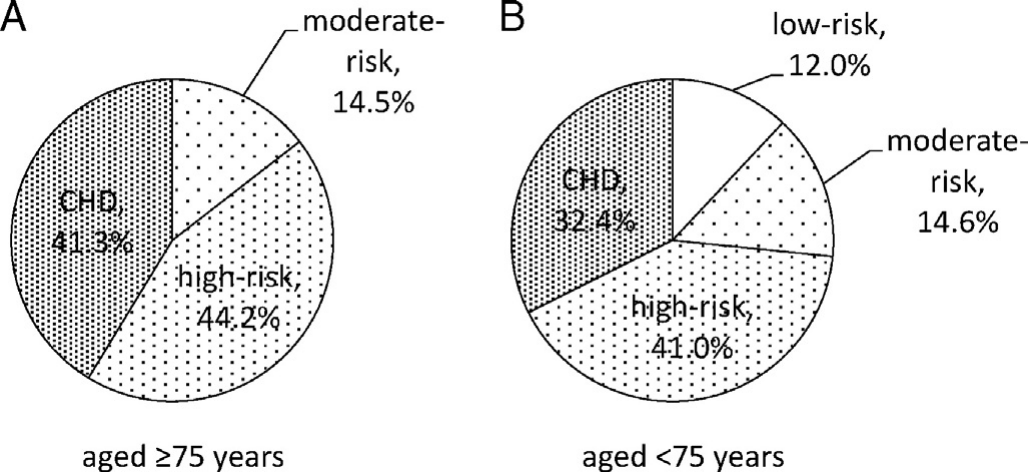
Comparison of risk stratification in patients between patients aged ≥ 75 (panel A) and < 75 (panel B).

Prevalence of CHD was 41.3% and 32.4% in patients aged ≥ 75 and < 75, respectively, (Fig. 1). In addition, none of the patients in patients aged ≥ 75 was stratified into low-risk, even though 12.0% of the patients were stratified into low-risk in patients aged < 75 (Fig. 1). Prevalence of primary prevention at high-risk patients was 44.2% and 41.0% in patients aged ≥ 75 and < 75, respectively, (Fig. 1).

### 3.5 Comparison of LDL-C and non-HDL-C target level achieving rates between the elderly and the non-elderly age groups

LDL-C and non-HDL-C target level achievement rates were 71.4% and 75.6% in patients aged ≥ 75, and these rates were 65.8% and 67.7%, respectively, in patients aged < 75. Thus, to our surprise, LDL-C and non-HDL-C target level achievement rates were significantly higher in patients aged ≥ 75 than those aged < 75 (*p* = 0.006 and *p* < 0.001, respectively).

**Fig. 2:**
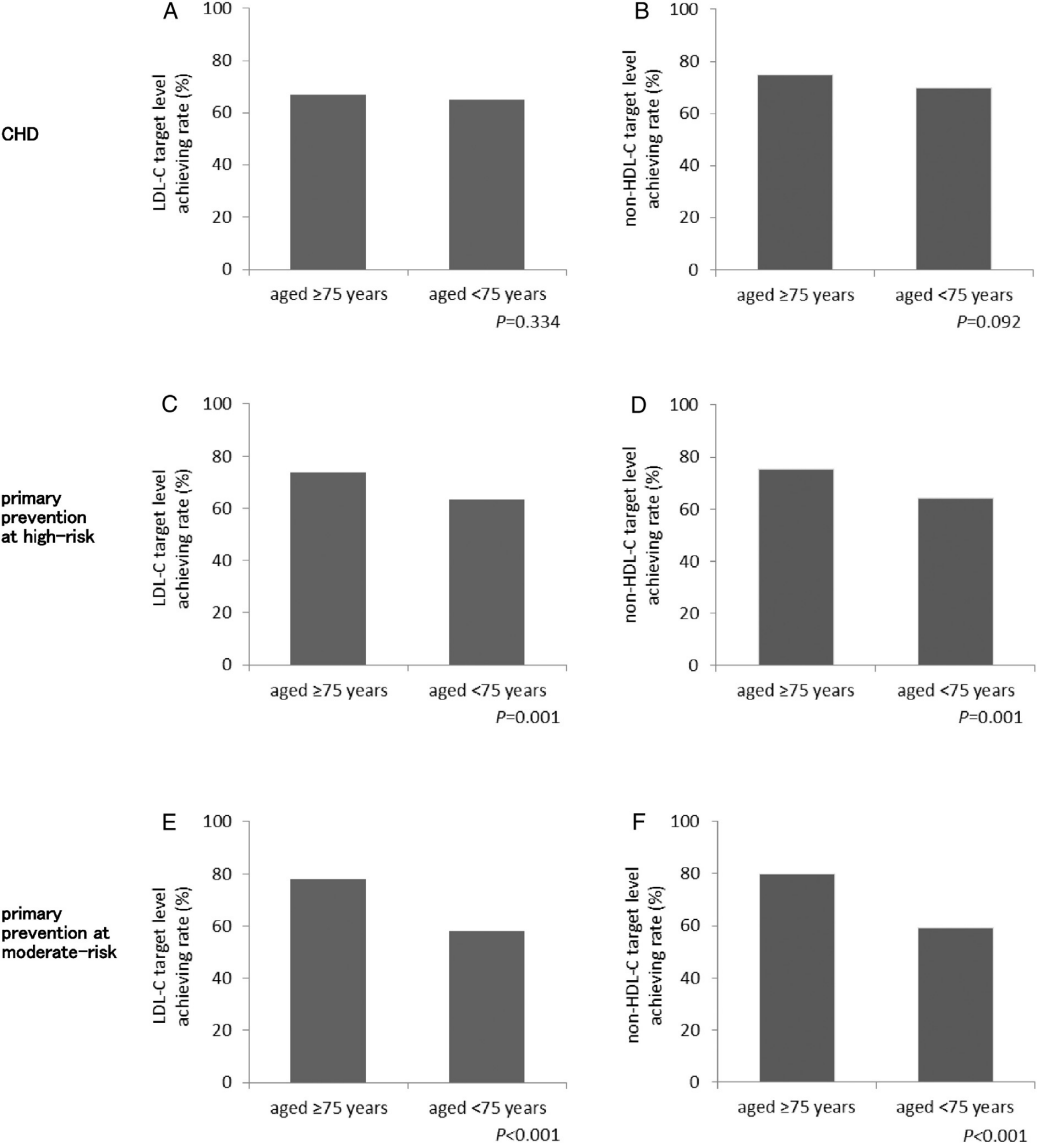
Comparison of LDL-C and non-HDL-C target level attainment between patients aged ≥ 75 and < 75 in various risk category subgroups. LDL-C (panels A, C, E) and non-HDL-C (panels B, D, F) target level achievement rates were compared between patients aged ≥ 75 and < 75 in various risk category subgroups, such as CHD (panels A, B), primary prevention at high-risk (panels C, D) and moderate-risk (panels E, F) subgroups. Values are expressed as percent. The *p* values were derived from χ^2^ statistics.

Lipid target level attainment was further evaluated in the different risk category subgroups. In CHD, LDL-C and non-HDL-C target level achievement rates were 66.9% and 74.7%, respectively, in patients aged ≥ 75, and they were 65.0% and 69.8%, respectively, in patients aged < 75. Thus, in CHD, LDL-C and non-HDL-C goal attainment was comparable between patients aged ≥ 75 and those aged < 75 (*p* = 0.334 and *p* = 0.092, respectively, Fig. 2A and B). In the primary prevention at high-risk subgroup, however, LDL-C and non-HDL-C goal attainment rates were higher in patients aged ≥ 75 (73.5% and 75.1%, respectively) than those aged < 75 (63.3% and 64.3%, respectively). These differences were statistically significant (*p* = 0.001 and *p* = 0.001, respectively, Fig. 2C and D). In the primary prevention at moderate-risk subgroup, LDL-C and non-HDL-C goal attainment also was better in patients aged ≥ 75 (77.9% and 79.8%, respectively) than those aged < 75 (58.1% and 59.1%, respectively). These differences also were statistically significant (*p* < 0.001, Fig. 2E and F). In CKD-G, LDL-C and non-HDL-C goal attainment rates were higher in patients aged ≥ 75 (70.2% and 69.5%, respectively) than those aged < 75 (58.1% and 59.1%, respectively), which were statistically significant differences (*p* = 0.018 and *p* = 0.036, respectively). In DM-G, in addition, they were 78.0% and 80.5%, respectively, in patients aged ≥ 75, and 69.3% and 71.5%, respectively, in those aged < 75, which were also significantly higher in patients aged ≥ 75 than those aged < 75 (*p* = 0.044 and *p* = 0.036 for LDL-C and non-HDL-C, respectively).

### 3.6 Comparison of lipid-lowering medication prescription rates between the elderly and the non-elderly age groups

The prescription rates of lipid-lowering drugs were compared between patients aged ≥ 75 and those aged < 75. Lipid-lowering medication prescription rates were 41.6% and 39.9% in patients aged ≥ 75 and < 75, respectively. Thus, lipid-lowering medication prescription rates appeared to be comparable (*p* = 0.248) between the two age groups.

**Fig. 3:**
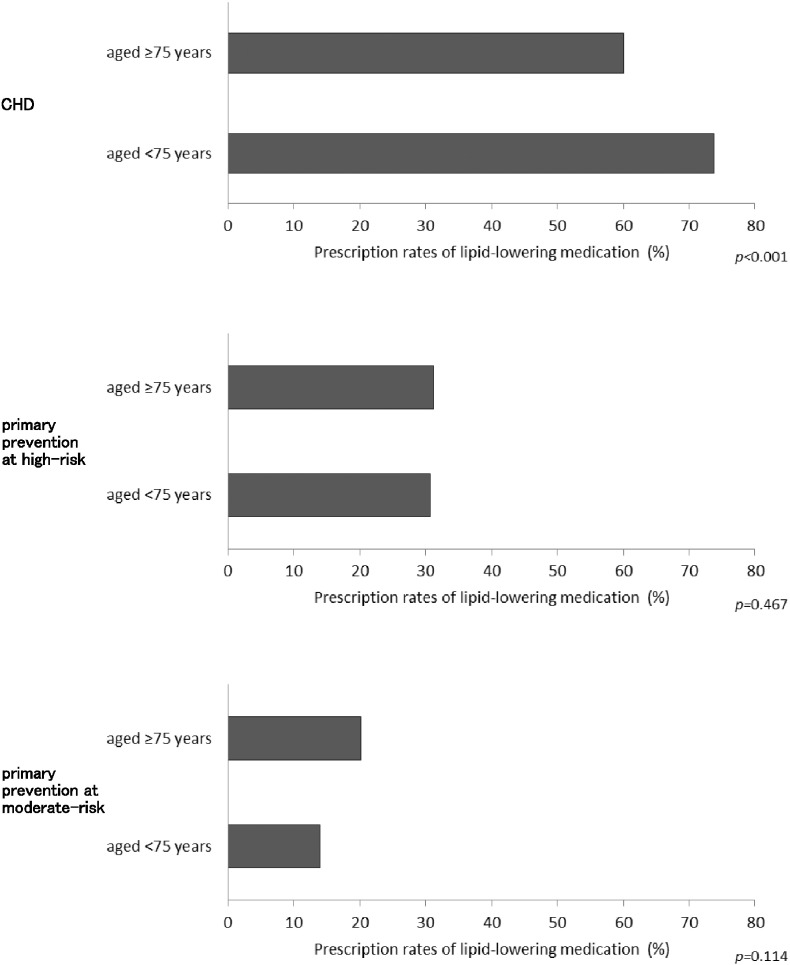
Comparison of lipid-lowering medication prescription rates between patients aged ≥ 75 and < 75 in various risk category subgroups Lipid-lowering medication prescription rates were compared between patients aged ≥ 75 and < 75 in various risk category subgroups, such as CHD (panel A), primary prevention at high-risk (panel B) and moderate-risk (panel C) subgroups. Values are expressed as percent. The *p* values were derived from χ^2^ statistics.

Lipid-lowering medication prescription rates were further compared between these two age groups in the different risk category subgroups. In CHD, lipid-lowering medication subscription rates were significantly lower in patients aged ≥ 75 (60.1%) than in those aged < 75 (73.8%, *p* < 0.001, Fig. 3A). In the primary prevention at high-risk subgroup, these rates were comparable between the two age groups. (31.2% and 30.7%, respectively, *p* = 0.467, Fig. 3B). In the primary prevention at moderate-risk subgroup, lipid-lowering medication subscription rates were also comparable between the two age groups (20.2% and 14.0% for patients aged ≥ 75 and < 75, respectively, *p* = 0.114, Fig. 3C). In CKD-G, they were 31.9% and 26.2% in patients aged ≥ 75 and < 75, respectively (*p* = 0.160). In DM-G, they were comparable between the two age groups (35.8% and 36.7%, respectively, *p* = 0.471). Thus, lipid-lowering medication was less frequently prescribed in patients aged ≥ 75 than those aged < 75 in the secondary prevention for CHD, and was almost equally prescribed in patients aged ≥ 75 and < 75 in the primary prevention at high-risk and moderate-risk subgroups, including CKD-G and DM-G. These results were quite different from what we had anticipated before this study.

In addition, contents of the lipid-lowering medication were compared between patients aged ≥ 75 and < 75. Prescription rates for drug combinations and monotherapies of lipid-lowering drugs were compared between two age groups in the total enrolled patients (Table 3) and in the patients with CHD (Table 4). The combination of statin plus EPA was more frequently prescribed in patients aged < 75 than in those aged ≥ 75, in the total enrolled patients (Table 3) as well as in those with CHD (Table 4). These differences were statistically significant (*p* = 0.002 and *p* = 0.001, respectively). In CHD, furthermore, statin monotherapy was also significantly more prevalent in patients aged < 75 than those aged ≥ 75 (*p* = 0.016, Table 4).

## 4 Discussion

Atherosclerosis is a continuous degenerative process, and its burden increases progressively with aging (Ulucam, 2012). Dyslipidemia is one of the most important risk factors in the development of atherosclerosis. According to the JAS2012-GL, patients aged ≥ 75 with dyslipidemia and primary prevention for CHD should be individually treated flexibly by the decisions of their attending physicians based upon the condition of each patient, such as accompanying other chronic diseases, frailty, drug tolerability, and social activities. On the other hand, patients aged 65–74 with dyslipidemia should be treated in the same way as those aged < 65 to achieve their serum lipid goals (Japan Atherosclerosis Society, 2014).

The PROSPER trial showed that three-year statin treatment in patients aged 70 to 82 years, including secondary prevention patients, decreased the risk of death from CHD plus nonfatal myocardial infarction by 19%, clearly demonstrating that intervention with statins may be indicated for the elderly. A decreased incidence of CHD was more clearly observed in men than in women, and in secondary prevention patients compare d to primary prevention patients; however, these differences were statistically insignificant (Shepherd et al., 2002). In addition, meta-analyses of the Cholesterol Treatment Trialists' (CTT) collaboration revealed that patients aged ≥ 75 tended to be less effective in the CHD risk reductions by statins than those aged < 75; however, they were not statistically significant differences (Baigent et al., 2010).

In the present study, the status of the serum lipid management in real-world clinical practice has been explored comparing the patients aged ≥ 75 to those aged < 75. To the best of our knowledge, this is the first report that was directed to the elderly patients (aged ≥ 75) comparing their serum lipid goal attainment rates to those in the non-elderly (aged < 75) patients. In fact, we had supposed that the LDL-C and non-HDL-C target level achievement rates in patients aged ≥ 75 may be lower than those aged < 75 in the primary prevention, and that they may be comparable in the secondary prevention patients, according to the guideline. Contrary to our expectation, the LDL-C and non-HDL-C target level achievement rates tended to be higher in the elderly (aged ≥ 75) patients than those aged < 75 in the primary prevention, although they are comparable between patients aged ≥ 75 and those aged < 75 in the secondary prevention. In patients aged ≥ 75 with CHD, lipid-lowering medication subscription rate was 60.1%, which was lower than that (73.8%) in patients aged < 75. They may result from the fact that lipid levels in patients aged ≥ 75 were lower than those in patients aged < 75 which appeared to be supported by previous studies (Ettinger et al., 1992, Schupf et al., 2005).

In the elderly, they more often have advanced presymptomatic atherosclerotic vascular lesions, which impose CHD events, than in the non-elderly subjects (Kannel, 2002). Elevated serum cholesterol is associated with greater number of CHD events (higher absolute risks) in the elderly than in the middle-aged or younger people. Consequently, reducing cholesterol concentration from high to low may well result in a greater overall reduction in new CHD events in the elderly population than in the middle-aged people, who generally have lower absolute risks (Gobal and Mehta, 2010). Therefore, although the relative risk reduction by lipid lowering may be smaller in the elderly than in the middle-aged subjects, the absolute risk reduction is greater in the elderly (Miettinen et al., 1997, Lewis et al., 1998, Heart Protection Study Collaborative Group, 2002) because of the higher baseline event rates (Berthold and Gouni-Berthold, 2011). At least, the JAS guideline does indicate that patients, who have already been taking and tolerating statins, should continue to take these drugs beyond 75 years of age (Japan Atherosclerosis Society, 2014).

In National Clinical Guideline in the UK, the Guideline Development Group members were aware that people aged 85 years or older have greater absolute risks of ACVD events, when compared with people aged below 85, and that they thus might have a greater likelihood of clinical benefits with statins (National Clinical Guideline Centre, 2014). Elderly people, in general, are more likely to have other comorbidities, poorer renal function, shorter life expectancy and other medication. Therefore, it would be recommended that statins should be prescribed with lower starting doses in the elderly (Stone et al., 2014, Reiner et al., 2011). There are very few data on cardiovascular outcomes in patients aged ≥ 75 with primary prevention for CHD. Therefore, Ezetimibe Lipid Lowering Trial on Prevention of Atherosclerosis in 75 or Older (EWTOPIA75 trial), using ezetimibe and powered for cerebrovascular and cardiovascular event endpoints, is currently ongoing in the primary prevention patients aged ≥ 75 with high LDL-C levels in Japan since 2009 (The Japan Geriatrics Society, n.d.).

Limitations of the present study may include the fact that this is a single-center retrospective cross-sectional study in patients who had been hospitalized in the Departments of Nephrology, Diabetes, Neurology, Respiratory Medicine and Cardiology and, therefore, results may not be generalizable to a broader spectrum of patients aged ≥ 75. In the primary prevention, serum lipid levels may be worse managed in subjects aged ≥ 75, if whole the outpatients, who had not been hospitalized, are included in this study. Especially, elderly subjects with sarcopenia or frailty would be much less frequently treated with lipid-lowering medication. Hence, future studies, including community-based residents with larger sample sizes may be needed to confirm the findings of the present study.

In conclusion, serum lipid goal attainment was comparable between the elderly and non-elderly patients with CHD, although the lipid-lowering drugs were less frequently prescribed in the elderly. Without CHD, it was significantly better in the elderly patients than the non-elderly subjects, although the lipid-lowering drug subscription rates were comparable between the elderly and non-elderly. Thus, baseline LDL-C and non-HDL-C levels before treatment with lipid-lowering drugs might have appeared higher in the non-elderly patients with and without CHD; therefore, their serum lipid management should be improved by more aggressive medical treatment according to the JAS-GL 2012.

## Conflict of interest statement

The authors declare no conflict of interest.

## Figures and Tables

**Table 1:** Characteristics of enrolled patients.

	Enrolled patients	Aged ≥ 75 years	Aged < 75 years	*p*
*Department*				< 0.001
Nephrology	100 (180)	27.8 (50)	72.2 (130)	< 0.05
Diabetes	100 (176)	21.0 (37)	79.0 (139)	< 0.01
Neurology	100 (41)	39.0 (16)	61.0 (25)	
Respiratory Medicine	100 (277)	44.8 (124)	55.2 (153)	< 0.01
Cardiology	100 (1314)	37.3 (490)	62.7 (824)	
Total	100 (1988)	36.1 (717)	63.9 (1271)	
Age, y	67.7 ± 0.3 (1988)	81.2 ± 0.2 (717)	60.0 ± 0.4 (1271)	
Mean body mass index (kg/m)^2^	23.1 ± 0.1 (1939/1988)	22.1 ± 0.1 (695/717)	23.7 ± 0.2 (1244/1271)	< 0.001
Gender				< 0.001
Male	64.4 (1280)	58.6 (420)	67.7 (860)	
Female	35.6 (708)	41.4 (297)	32.3 (411)	
Risk factors				
HT	57.8 (1150)	62.6 (449)	55.2 (701)	0.001
CKD	40.4 (804)	52.7 (378)	33.5(426)	< 0.001
DM	36.1 (718)	34.3 (246)	37.1 (472)	0.113
CHD	35.6 (708)	41.3 (296)	32.4 (412)	< 0.001

*Lipid profiles*				
LDL-C (mg/dL)	100.3 ± 0.7 (1981/1988)	94.3 ± 1.1 (715/717)	103.7 ± 0.9 (1266/1271)	< 0.001
TG (mg/dL)	132.7 ± 1.8 (1986/1988)	118.4 ± 2.5 (716/717)	140.7 ± 2.5 (1270/1271)	< 0.001
HDL-C (mg/dL)	51.3 ± 0.4 (1988)	49.6 ± 0.6 (717)	52.3 ± 0.5 (1271)	< 0.001
non-HDL-C (mg/dL)	124.9 ± 0.9 (1831/1988)	116.8 ± 1.3 (679/717)	129.7 ± 1.1 (1152/1271)	< 0.001
eGFR (mL/min/1.73 m^2^ )	64.9 ± 1.0 (1974/1988)	55.4 ± 0.9 (715/717)	70.3 ± 1.4 (1259/1271)	< 0.001

Values are expressed as percent (n) or mean ± SEM (n). HT, hypertension; CKD, chronic kidney disease, DM, diabetes mellitus; LDL-C, LDL cholesterol; TG, triglycerides; HDL-C, HDL cholesterol; non-HDL-C, non-HDL cholesterol; eGFR, estimated glomerular filtration rate.

**Table 2:** Comparison of lipid profiles between male and female.

	Male	Female	*P*
LDL-C (mg/dL)	97.2 ± 0.9 (1273/1280)	106.0 ± 1.3 (708)	< 0.001
TG (mg/dL)	137.8 ± 2.4 (1279/1280)	123.4 ± 2.6 (707/708)	< 0.001
HDL-C (mg/dL)	48.7 ± 0.4 (1280)	56.2 ± 0.6 (708)	< 0.001
non-HDL-C (mg/dL)	122.0 ± 1.0 (1165/1280)	130.1 ± 1.6 (666/708)	< 0.001

Values are expressed as mean ± SEM (n).

**Table 3:** Prescription rates for drug combinations and monotherapies of lipid-lowering medications in total enrolled patients.

		Aged ≥ 75 years	Aged < 75 years	*p*
Quadruple therapy	Statin, fibrate, ezetimibe, EPA	0.1 (1)	0.0 (0)	0.361
Triple therapy	Statin, ezetimibe, resin	0.0 (0)	0.1 (1)	0.639
Statin, ezetimibe, EPA	0.3 (2)	0.4 ()	0.508
Statin, ezetimibe, nicotinic acid	0.3 (2)	0.0 (0)	0.130
Dual therapy	Statin, fibrate	0.1 (1)	0.2 (2)	0.704
Statin, ezetimibe	1.3 (9)	1.2 (15)	0.519
Statin, EPA	0.7 (5)	2.5 (32)	0.002
Statin, nicotinic acid	0.6 (4)	0.7 (9)	0.468
Fibrate, ezetimibe	0.0 (0)	0.1 (1)	0.639
Ezetimibe, EPA	0.1 (1)	0.1 (1)	0.591
Monotherapy	Statin	36.3 (260)	33.3 (423)	0.098
Fibrate	0.3 (2)	0.6 (7)	0.312
Ezetimibe	0.3 (2)	0.2 (3)	0.592
EPA	1.0 (7)	0.3 (4)	0.058
Nicotinic acid	0.3 (2)	0.3 (4)	0.626
Without medication		58.4 (419)	60.1 (764)	0.248

Values are expressed as percent (n).

**Table 4:** Prescription rates for drug combinations and monotherapies of lipid-lowering medications in CHD.

		Aged ≥ 75 years	Aged < 75 years	*p*
Quadruple therapy	Statin, fibrate, ezetimibe, EPA	0.3 (1)	0.0 (0)	0.418
Triple therapy	Statin, ezetimibe, resin	0.0 (0)	0.0 (0)	
Statin, ezetimibe, EPA	0.7 (2)	1.2 (5)	0.380
Statin, ezetimibe, nicotinic acid	0.3 (1)	0.0 (0)	0.418
Dual therapy	Statin, fibrate	0.3 (1)	0.0 (0)	0.418
Statin, ezetimibe	2.4 (7)	2.9 (12)	0.422
Statin, EPA	1.7 (5)	6.6 (27)	0.001
Statin, nicotinic acid	0.3 (1)	1.2 (5)	0.205
Fibrate, ezetimibe	0.0 (0)	0.0 (0)	
Ezetimibe, EPA	0.0 (0)	0.0 (0)	
Monotherapy	Statin	52.0 (154)	60.4 (249)	0.016
Fibrate	0.3 (1)	1.2 (5)	0.205
Ezetimibe	0.3 (1)	0.0 (0)	0.418
EPA	1.0 (3)	0.2 (1)	0.200
Nicotinic acid	0.3 (1)	0.0 (0)	0.418
Without medication		39.9 (118)	26.2 (108)	< 0.001

Values are expressed as percent (n).
